# Pharmaceutical research in paediatric populations and the new EU Paediatric Legislation: an industry perspective

**DOI:** 10.1186/1753-2000-2-38

**Published:** 2008-12-08

**Authors:** Philippe Auby

**Affiliations:** 1 Director, International Clinical Research – Paediatric Neuro-Psychiatry, Lundbeck SAS, 37 avenue Pierre 1er de Serbie, 75008 Paris, France

## Abstract

A large proportion of medicines used in children are prescribed off-label, and children have often been denied access to new or innovative medications. Because such situation is unethical, the need to obtain paediatric information for medicines used in children seems nowadays a matter of consensus on a global basis. Based on this, it was clear in EU, like what has happened in the US, that there was a need for a legal obligation for Pharmaceutical Companies to perform studies. This new European Paediatric Regulation that entered into force in 2007 opens a new era of European drug regulatory history and will offer a major opportunity to improve children's health through advancements in research by providing a new framework for evaluating the efficacy and safety of medicines for children. But, paediatric development remains challenging and the hurdles of conducting research in paediatric population are numerous. The article presents the new European Paediatric Regulation, illustrates its rationale through paediatric psychopharmacology, and discusses some of its consequences on paediatric research from an industry perspective. Recommendations for further international collaboration are also suggested to make global paediatric development plans.

## Background

A large proportion of medicines used in children are prescribed outside the terms of the drug license i.e. off-label, which can place children at a direct risk of under- or overdosing and a delayed risk of long-term adverse effects. Many generations of paediatricians and other physicians have learned to live with the situation [1].

This off-label use of medicines in children has however been an increasing concern over the last decade leading to recognize that such situation was unethical as children have not access to medications properly assessed.

### US perspective

In 1994, the United States implemented the "Pediatric Labeling Rule" which paved the way for legislation aimed at producing drugs for children. But the first critical paediatric legislative initiative in the US is the Food and Drug Administration Modernization Act that in 1997 provided an incentive for Pharmaceutical Companies to study products for which there would be a health benefit in the paediatric population. This legislation enacted a voluntary process where FDA would define the products which needed paediatric studies, outline the necessary studies, and issue sponsors a Paediatric Written Request (PWR). If the Pharmaceutical Companies submitted studies responding to the PWR, six additional months of marketing exclusivity were received. This process has been the main legislative initiative that has moved paediatric drug development in the US. However some gaps were identified in the FDA's 2001 Report to Congress, e.g. that this incentive legislation was only working for some products, and were partially addressed by the Best Pharmaceuticals for Children in 2002 (BPCA). This Act renewed the exclusivity incentives, created an off-patent process involving government contracts for paediatric studies, and mandated public disclosure of the study results. In 1997, the FDA proposed, and in 1998 finalized the Pediatric Rule. In December 2003, the Pediatric Research Equity Act (PREA) was enacted, putting into legislation most components of the Pediatric Rule for instance requiring paediatric assessment for certain applications unless waived or deferred.

These critical steps taken in the late 1990's and early 2000's in the US were amended and reauthorized in 2007 as Pediatric Research Equity Act of 2007 and Best Pharmaceuticals for Children Act of 2007. Both of these are clearly designed to encourage more paediatric research and more development of paediatric medicines.

### EU perspective

In 2000 in the European Union, fifty per cent or more of medicines used in children have never been studied in this population, but only in adults, and not necessarily in the same indication (or the same disease) [2]. Even if paediatric clinical studies have been performed in some cases, very few medicinal products used have a paediatric indication and a defined posology, and even less a formulation allowing the administration to young children.

The need for more studies to obtain paediatric information for medicines used in children seems nowadays a matter of consensus on a global basis [3]. Based on this, it was clear in EU that there was a need for a legal obligation for Pharmaceutical Companies to perform studies if they intended to develop medicines for use in the paediatric population [3].

## The New European Paediatric Legislation

A new legislation governing the development and authorisation of medicines for paediatric use (children and adolescents aged 0 to 17 years) was introduced in the European Union (EU) in December 2006 and entered into force in January 2007 [4]. Like the US paediatric legislation, the goals of the EU legislation are the same i.e. to improve children's health through advancements in research and to provide a new framework for evaluating the efficacy and safety of medicines for children. However, unlike in the US, the EU legislation is leading to more profound and faster changes in the field of paediatric development in Europe; more profound as unlike in the US paediatric development will become mandatory in EU for all new medicinal products in development unless a waiver is granted, and as Pharmaceutical Companies have to send a paediatric investigation or development plan as early as the end of pharmacokinetic studies in adults; faster as the changes are occurring in EU in a shorter period of time. Actually, the timelines of obligation for Pharmaceutical Companies are that: 18 months from entry into force (July 2008), applications for new marketing authorisation applications (new products) should contain results of studies conducted in compliance with agreed Paediatric Investigation Plan (PIP) unless waiver or deferral; 24 months from entry into force (January 2009), application for new indications, new routes of administration or new pharmaceutical forms should contain results of studies in compliance with agreed PIP unless waiver or deferral.

The aim of this new regulation is to improve the health of the children of Europe, by:

- Increasing high quality research into medicines for them,

- Promoting the development and authorisation of such medicines, and over time, ensuring that the majority of medicines used by children are specifically authorized for such use,

- Improving the availability of high quality information on medicines designed for children.

This regulation makes paediatric development as an obligation in the EU, with the following key points:

- Creation of a European Paediatric Committee (PDCO), replacing the former Paediatric Expert Group. The PDCO first met in July 2007. The committee is composed of experts with competence in the development and assessment of all aspects of paediatric medicinal products and the EMEA Executive Director must ensure that the final composition of the PDCO covers all related relevant disciplines: 5 members (and alternates) from the Committee for Medicinal Products for Human Use (CHMP), 1 member (and alternates) from each Member State not represented via CHMP membership, 6 members (and alternates) appointed by the European Commission representing healthcare professionals (3) and patients' organisations (3). The PDCO has been operational with 27 members, even before finalisation of the appointment by the European Commission of the further 6 members representing healthcare professionals and patients' associations.

- Submission of Paediatric Investigation Plan (PIP) at availability of adult pharmacokinetic studies, i.e. at an early phase of the development of a new compound. A PIP is a development plan aimed at ensuring that the necessary data are obtained through studies in children, when it is safe to do so, to support the authorisation of the medicine for children. The plan should be submitted by Pharmaceutical Companies to the PDCO Committee, which is responsible for agreement or refusal of the plan.

- Paediatric data is mandatory for all regulatory submissions for new products and for products still on patent in case of line extension (unless waivers or deferrals) according to a PIP agreed upon by the PDCO.

- PIP reflects the development plan on clinical, non-clinical and technical aspects including timelines and covers all existing or planned (adult) indications and dosage forms (including specific age-appropriate paediatric formulation or route of administration if necessary). Applications should also cover all subsets (according to ICH E11) of the paediatric population from birth to adolescence. The plan clearly defines the timing of studies in children compared to adults. In some cases, studies will be deferred until after the studies in adults have been conducted, to ensure that research with children is done only when it is safe and ethical to do so.

- PIP can be amended and is binding on the company.

- Reward for studies conducted can result in a 6-month patent extension.

This should be achieved without subjecting children to unnecessary clinical trials and should not delay the authorisation of medicines for use in adults.

In certain circumstances, the requirement to submit a paediatric investigation plan can be waived for specific medicinal products or classes of medicinal products that:

- Are likely to be ineffective or unsafe in part or all of the paediatric population,

- Are intended for conditions that occur only in adult populations e.g. Alzheimer's disease,

- Do not represent a significant therapeutic benefit over existing treatments for paediatric patients.

In accordance with the Paediatric Regulation, the PDCO has adopted a list of conditions that occur only in adult populations. All classes of medicinal products intended to treat these conditions will therefore be exempt from the requirement for a paediatric investigation plan.

## Discussion

### The example of child psychopharmacology

If the therapeutic effects of amphetamines in hyperactive children were first described in 1937, thus, preceding the major discoveries of adult psychopharmacology, since this, little innovation has occurred in paediatric psychopharmacology [5]. Furthermore, while progress in the recognition and treatment of mental disorders in childhood and adolescence has been accomplished, the task of turning basic research findings into clinically useful applications still remains in front of us [6].

Actually, only few psychotropic medications are approved for use in the paediatric population. However, it has become increasingly common to use these medications to treat a variety of mental health disorders in children and adolescents but this has not constantly been supported by rigorous scientific data. A study of the prescribing trends in nine countries between the years 2000 and 2002, evidenced that the increase in psychotropic prescribing in children was not only confined in the USA and UK but is also evident in the 7 other examined countries (Argentina, Brazil, Canada, France, Germany, Mexico & Spain) [7].

The questions related to the use of Selective Serotonin Reuptake Inhibitors (SSRIs) in Paediatric Major Depressive Disorder (MDD) can provide an opportunistic example of where paediatric pharmaceutical research can improve. The official recognition of depression in children and adolescents in Europe took place in 1971, when the Union of European Pedopsychiatrists recognised and addressed the needs of depressed children and adolescents by declaring that depression is an important illness that constitutes a significant proportion of mental disorders in children and adolescents [8]. During the 1980s, the arrival of the SSRIs, which resulted in far less side effects than tricyclics or monoamine oxidase inhibitors, was viewed as an important step in the treatment of affective disorders, first in adults and then in children and adolescents [9]. Simultaneously, the literature on the treatment of MDD in children and adolescents has significantly grown since the introduction of the SSRIs. Although the exact mechanism of action responsible for the therapeutic effects of many psychotropics remains unknown, the basic biochemical activity of these medications is generally considered to be similar across all ages [10]. In both paediatric and adult patients, SSRIs block the reuptake of serotonin and their antidepressant effect has been found to be associated with the degree of inhibition of the serotonin transporter in platelets [11]. However, it still remains to be proven whether SSRIs that are efficacious in adults are also efficacious in treating MDD in children and adolescents [12]. Most of the clinical studies did not demonstrate superiority of active treatment when compared to placebo as: only fluoxetine was repetitively superior to placebo on primary outcome measures; studies of citalopram, sertraline and escitalopram recently [13], have also shown superiority over placebo on primary outcome measures; studies of paroxetine, venlafaxine, mirtazapine, nefazodone, and tricyclics, have not demonstrated superiority of any of these pharmacological treatments over placebo on the primary efficacy measures [9,14-16]. At present, only fluoxetine is approved in EU and US for paediatric MDD.

The reasons why many of these studies have failed remains unclear. Although some of these antidepressants may not be beneficial (like probably tricyclics), methodological considerations have been raised, among them dosing issues should be carefully evaluated. Extrapolation from adult data is definitively insufficient. Some authors hypothesized that inaccurate dosing parameters may have participated in the negative outcome of the studies of antidepressants in paediatric patients with MDD [17].

Key parameters of dosing that should be evaluated include identifying an appropriate total daily dose and determining how frequently the medication needs to be administered every day. If a medication is not dosed properly, clinical efficacy might no be detected during a clinical trial [17]. The selection of doses in paediatric patients requires a consideration of pharmacokinetic parameters and warrants specific studies in children and adolescents to establish benefits and risks during drug development [18], deemed as a pivotal aspect of paediatric drug development. Reviewing the pharmacokinetic (PK) studies performed in children and adolescents with SSRIs, R. Findling *et al. *in 2006 concluded that in many instances, the dosing strategies that have been employed in the placebo-controlled efficacy studies in juvenile MDD were not supported by the data available from PK studies [17]. Therefore, these authors emphasize the need to develop evidence-based dosing strategies before studying any drug in paediatric population as medication dosing regimens may have contributed to both failure to demonstrate efficacy and safety and tolerability concerns [17]. Reviewing the paediatric randomized controlled MDD trials, Moreno *et al. *reached a similar conclusion: as antidepressants have two to three times shorter half-lives in youngsters, they need to be administered more often than to adults to avoid withdrawal symptoms between doses that can be wrongly interpreted as the absence of an adequate response with the exception of fluoxetine, which has a longer half-life [12]. Consequently PK and dose ranging studies are needed to inform the design of definitive efficacy trials. But such type of paediatric studies remain difficult to perform and alternative like modeling are developed as they are ethically challenging mainly due to the fact that such research does not offer a prospect of direct benefit.

### The new EU Paediatric Regulation: an ongoing learning process

Contrary to what has happened in the US, the EU paediatric legislation is leading to more dramatic and faster changes in a still moving and complex environment. The legislation entered into force in January 2007, the PDCO first met in July 2007, and the Commission Guideline on format and content of Paediatric Investigation Plan was on a draft format until September 2008 when the final version was published by the European Commission, implying that all stakeholders had and still will have to work together and interact to overcome the challenges of this new regulation.

Numerous aspects of this new process will lead to interesting interactions and future developments.

The EU paediatric legislation does not make any difference between products already on the market and drugs in development. The transition period does not allow enough flexibility to take into account in some cases, specific product patent timelines meaning that paediatric development may not be possible for some products still on patent. It is too early to draw any clear conclusion but the fact that after one year almost two third of the applications are for medicines that are not yet authorised or approved in EU (PDCO first anniversary) seems to be in favour of this concern. It could be wished that for new products, there would be more opportunities to interact with the PDCO. Therefore, it could be of interest to offer further opportunities of direct interactions between the PDCO and the Pharmaceutical Companies as improving the communication around the common goal to develop better medicines for children between the PDCO and the Pharmaceutical Companies can only be beneficial.

### Towards a new drug development paradigm?

It is too early to determine how the new EU paediatric regulation will affect the way in which drugs are developed. For Pharmaceutical Companies, the requirements resulting from the paediatric regulation would probably lead to a new drug development paradigm integrating paediatric considerations extremely early in the process of developing a new chemical entity.

If the timing of PIP submission i.e. the end of adult PK studies can be interpretated as favouring such paradigm, more emphasis on integrated paediatric and adult development could have been suggested in the Commission Guideline. Such new drug development paradigm however will pose specific ethical and scientific challenges.

The example of atomoxetine development can be useful, as it has heavily been influenced by US paediatric regulation and guidance from the FDA, also showing that new and integrated adult and paediatric models can be achieved [19].

### Need for a review of EMEA guidelines for paediatric considerations

The EMEA guidelines for psychiatric conditions (mainly for efficacy) will need to be revised with specific paediatric considerations. These guidelines provide already some clear guidance as they confirm the existence of numerous paediatric conditions in different age groups (according to ICH E 11 [20]) but the methodological sections lack paediatric specificities. The first paediatric EMEA guideline under development will be for ADHD and should offer an integrated adult/paediatric development.

Two specific aspects can illustrate this question such the use of placebo in children and adolescents and the question of comorbidity.

If from a scientific point of view, randomised double-blind comparisons versus placebo are often preferable to permit adequate evaluation of efficacy and safety/tolerability, the use of placebo raises ethical concerns potentially leading to different opinions between Health Authorities and Ethics Committees. Ethical requirements must be taken into consideration when designing paediatric protocols and PIPs and paediatric protocols cannot simply be mimic adult protocols. For instance, rescue treatment and escape procedures should always be considered in paediatric trials: rescue refers to treatment that may be given on top of trial medications to avoid danger or distress, for example pain treatment, as soon as the patient reaches a defined level; escape refers to prompt removal of subjects whose clinical status worsens or fails to improve to a defined level in a trial [21].

Comorbidity is not accepted in the current EMEA guidelines, and the patients to be included in the trials should have only one specific disease (e.g. patients with MDD and with no anxiety disorders). However, it is well established in child and adolescent psychiatry that comorbidity is the rule rather than the exception [22]: clinical and epidemiological investigations have revealed that 40%–70% of depressed children and adolescents have comorbid psychiatric disorders and that at least 20%–50% have two or more comorbid diagnoses [8].

### Limited EU paediatric experience

Compared to the US, the EU experience in paediatric research is less extensive. In the field of child and adolescent psychopharmacology, the majority of publications and studies are coming from the US. Reviewing 27 placebo-controlled trials assessing the use of antidepressant medications among more than 4400 children and adolescents published between January 1998 and July 2006 in Medline, Apter et al. reported that 23 out of 27 were conducted solely in the US and only 3 were done partly in European countries [23]. This new legislation will help developing an EU network of potential investigators in child and adolescent psychiatry, emphasizing that identification and training of new research centers will also have to take place. However it will be necessary to take into account the public perception of paediatric research in Europe and the awareness of Ethics Committees. Currently, the European Commission's Guideline on the PIP does not take into account feasibility issues. If this is understandable, such feasibility potential issues or concerns will be translated to facts e.g. geographic localisation of the study when the first studies part of the PIPs will be recruiting and may lead to PIPs amendments.

### Towards a global paediatric development plan

Another major challenge will be to ensure as much as possible global paediatric development mainly for EU and US (keeping however in mind that other countries are also following this path of paediatric legislation), working ideally on common study designs in order to avoid unnecessary duplication of studies and expose children to undue risks. In June 2007, the US Food and Drug Administration (FDA), the European Commission (EC), and the European Medicines Agency (EMEA) have agreed to expand their current cooperative activities in several important areas including paediatrics. Numerous scientific issues offer an opportunity to seek a consensus between EU and US like for instance recommendations concerning the use of placebo or active comparators in paediatric psychopharmacology. At present, the FDA and the EMEA already work together, having monthly teleconferences, exchanging information on paediatric development. Both the EMEA and FDA are committed to develop a framework:

- To facilitate regular exchange of scientific and ethical issues and other information on paediatric development programmes in Europe and the US to avoid exposing children to unnecessary trials.

- To aim at global paediatric development plans based on scientific grounds and compatible for both Agencies.

However as the current different legal/regulatory requirements may prevent receiving identical applications for paediatric development plans, it would be of paramount importance to explore new areas of transatlantic regulatory cooperation and further strengthen such collaboration by developing a common process between FDA and EMEA, aiming for a global paediatric plan. A possible start towards global paediatric development could be to make Pediatric Written Requests and Paediatric Investigation Plans compatible; for instance considering the possibility of potentially amending the PWR or PIP depending on the feedback or requests of the other Agency and even incorporating such possibility in both regulations could offer an opportunity to make paediatric research more effective.

### Financial aspect of paediatric development

Finally, the financial aspect of paediatric development cannot be eluded and its impact on Pharmaceutical Companies will have to be assessed. In 2007, before the US paediatric legislation was renewed, Li et al [24] examined the returns on investment of completing paediatric exclusivity and demonstrated that the distribution of net economic return for 6 months of exclusivity varied substantially among products, being very positive for blockbusters but being also potentially negative in some cases. They concluded that the Pediatric Exclusivity Program overcompensates blockbuster products for performing clinical trials in children. There is a concern that, if paediatric development is more difficult and expensive than anticipated, about what could be the potential risk on research in Europe for primarily EU companies, especially for small or medium size companies.

## Conclusion

The European Paediatric Regulation is a major achievement and opens a new era of European drug regulatory history. Children have often been denied access to new or innovative medications and paediatric development still depends on the outcome of the adult development. This Regulation offers a major opportunity to improve children's health. But, paediatric development remains challenging and the hurdles of conducting research in paediatric population are numerous including 'moral' and ethical issues, scientific issues, practical issues and finally financial issues. Therefore as a shared responsibility among companies, regulatory authorities, health professionals, and society as a whole (ICH E-11), it is through the lessons learned during the implementation of this new legislation and the numerous dialogues that will result, that changes will occur, promoting paediatric research. Clearly further regulatory and scientific international collaborations are warranted to favour global paediatric development plans in order to federate efforts and initiatives, and consequently make paediatric research more effective and efficient. Ultimately, it is through well-conducted ethical and quality research that children and adolescents will gain access to new medications and receive safe and optimal drug therapy.

## Competing interests

PA is an employee of Lundbeck SAS. This article is based on a personal presentation made in Berlin in 2008 at the Conference of the Europäische Akademie: "Clinical Research in Vulnerable Populations" and may not necessary express the views and opinion of H. Lundbeck A/S or its affiliates.
